# Primary Pulmonary Lymphoma and Synchronous Cecal Adenocarcinoma: A Rare Occurrence

**DOI:** 10.7759/cureus.944

**Published:** 2016-12-27

**Authors:** Amina Saqib, Uroosa Ibrahim, Rabih Maroun

**Affiliations:** 1 Pulmonary/Critical Care, Staten Island University Hospital; 2 Department of Hematology/Oncology, Staten Island University Hospital

**Keywords:** primary pulmonary lymphoma, adenocarcinoma, synchronous tumor, non-hodgkin cell lymphoma, pet/ct

## Abstract

In the era of extensive imaging and increasing indications for performing PET-CT scans, the recognition of synchronous tumors may be greater than before. However, the majority of these tumors are seen to occur in the same organ system, likely because of sharing similar pathogenic mechanisms. Synchronous lung cancers of similar or differing histologies have been reported. Primary pulmonary lymphoma, which is a rare form of non-Hodgkin’s lymphoma, has also been seen with a synchronous primary lung cancer. However, we report a case of a 56-year-old male diagnosed with primary pulmonary lymphoma and on PET-CT imaging, found to have a cecal lesion, the biopsy of which showed adenocarcinoma. We discuss the incidence of the co-existence of multiple tumors, the pathogenic mechanisms involved, and approaches to the management of these rare clinical scenarios.

## Introduction

Synchronous cancers, although rare, are usually seen within the same organ system or areas exposed to a carcinogen, such as the lung or the gastrointestinal tract. Among lung cancers, synchronous tumors are seen to arise on the same side of the lung and are usually of similar histology. Primary pulmonary lymphoma (PPL) is an extremely rare form of non-Hodgkin’s lymphoma arising from mucosa-associated lymphoid tissue (MALT). Although PPL with another primary lung cancer has been reported [1], our case is the first of its kind with PPL and a synchronous distant primary cancer, i.e., adenocarcinoma of the cecum. We discuss the incidence of synchronous malignancies, the role of imaging in diagnosis, and the implication of co-occurrence on the management of these rare scenarios.

## Case presentation

A 56-year-old male presented to his primary care physician complaining of chest pain. The patient was a non-smoker with a past history of dyslipidemia, hypertension, and chronic obstructive pulmonary disease (COPD). Physical examination was normal. A chest x-ray revealed a small opacity in the right middle lobe, and a computed tomography (CT) scan of the chest showed bronchiectasis and atelectasis in the right middle lobe with acute cutoff of the right middle lobe bronchus. The patient was thereafter referred to our pulmonary office. A bronchoalveolar lavage (BAL) was performed which was negative for the presence of malignant cells. Obscuring blood was seen along with acute inflammation. Stains and cultures for acid-fast bacilli (AFB), fungi and *Pneumocystis jiroveci* were negative. A bacterial culture showed growth of *Staphylococcus aureus, *and the patient was treated with Levaquin.

An endobronchial biopsy of the right middle lobe lesion was performed which showed the presence of PAX-5 positive B-cells, and CD3 and CD5 positive T-cells. CD20 and CD43 stains were non-contributory because of crush artifact. BCL-6 demonstrated scattered staining. BCL-1 was negative, and many cells demonstrated staining for Ki-67. Since a definite diagnosis could not be reached, a repeat BAL and biopsy of the right middle lobe endobronchial lesion was performed. The brushings were negative for malignant cells with the presence of histiocytes and benign squamous cells. The CD4 to CD8 T-cell ratio was 0.67:1. An ultrasound-guided fine needle aspiration (FNA) biopsy of the right hilar lymph node did not reveal any evidence of malignancy. Mycology culture showed scant growth of *Candida glabrata*. The right middle lobe biopsy showed a clonal lambda (dim) B-cell population expressing CD10. Hence, a diagnosis of primary pulmonary lymphoma was made.

**Figure 1:**
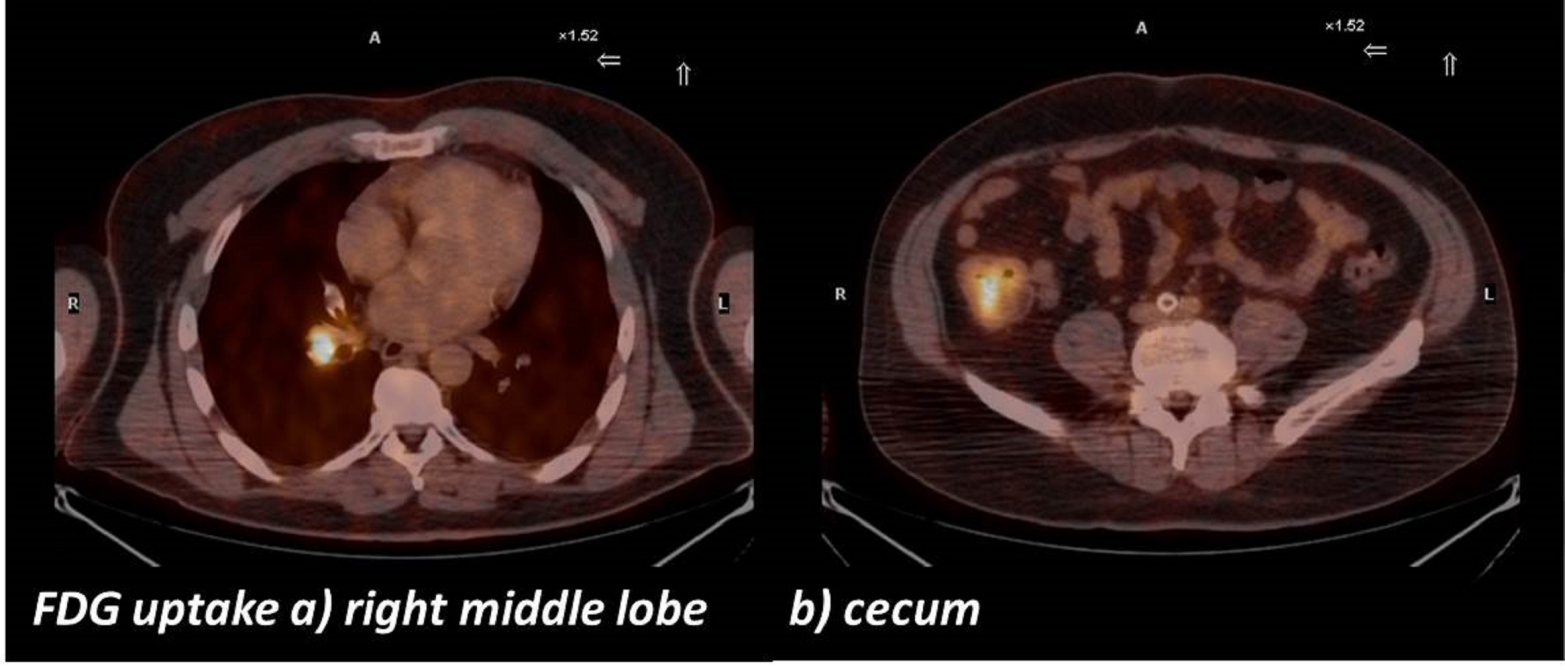
PET Scan Images FDG-uptake is shown in the a) right middle lobe, and b) the cecum.

A PET-CT scan performed to evaluate for any other sites of involvement revealed abnormal increased uptake in the right middle lobe bronchus with an SUV of 7.5, and focal increased uptake in the right hilar lymph node measuring 1.4 cm x 1.7 cm with an SUV of 17.3. In addition, focal increased uptake was also seen in the cecum with an SUV of 9.1 (Figure 1).

A colonoscopy with cecal biopsy showed adenocarcinoma, and the patient was referred to medical and surgical oncology for further management.

The patient agreed to participate and was explained the nature and objectives of this study, and informed consent was formally obtained. No reference to the patient's identity was made at any stage during data analysis or in the report.

## Discussion

Multiple primary cancers are a known occurrence and have long been studied in order to determine similar pathogenic mechanisms to guide a specific therapy. However, these cancers usually occur in the same organ or organ system, such as multiple pulmonary tumors or gastrointestinal tumors. This presentation of shared timing is termed synchronous as opposed to metachronous, wherein cancers of the same or different systems develop at intervals [2-3]. The concept of ‘field cancerization’ is postulated to predispose patients to synchronous tumors whereby shared risk factors cause tumors that are spatially separated, such as smoking for lung cancer. Similarly, synchronous esophageal and colon cancers occurring at an incidence of 2.9%, share the risk factors of smoking and alcohol intake, and the protective factor of vegetable and fruit intake [4].

PPL is a rare form of lymphoma with an incidence of 0.4%. It is a form of extranodal B-cell lymphoma, and about 70% to 80% are thought to arise from mucosa-associated lymphoid tissue. About 2% are Hodgkin’s lymphoma. In order for a diagnosis of PPL to be made, extrapulmonary disease must be ruled out. About two-thirds of patients present with symptoms being present for a mean duration of five months. Various radiologic manifestations have been described including a patchy opacity, single or multiple nodules, mediastinal mass, or pleural effusions. Unilateral and bilateral cases have been reported. Given the indolent nature of the disease, local treatment results in excellent outcomes [5].

Zheng et al., reported the only case of synchronous PPL with pulmonary adenocarcinoma presenting as two FDG-avid lesions in the middle and lower lobes of the right lung which was treated with a right middle lobectomy, a partial lower lobectomy, and an adjuvant pemetrexed [1]. Our case is extremely unique in terms of presenting with PPL and a synchronous distant primary lesion of adenocarcinoma of the cecum that came to attention from a PET scan done for the staging of the PPL.

A decision of whether or not to pursue a lesion seen on imaging performed for another reason is a difficult one to make. The imaging characteristics, clinical information, and location of the lesion can guide further management. With PET-CT imaging being more widely available and used, distant synchronous pathologies may be detected incidentally. Chun-Sing et al., examined 1,522 PET-CT scans performed at their institution and found that 68 patients had synchronous lesions [6]. Of those, 11.7% had a pathologic conformation of a second primary, one of which was benign. Hence, among all patients undergoing a PET-CT scan, the incidence of synchronous tumors was 0.5% [7-8].

In cases of synchronous cancers, the tumor biology and prognosis of one primary can have an impact on the management of the other. For instance, the more indolent of the two entities will be treated less aggressively if treatment strategies are not similar and lead to added toxicity. On the other hand, a definite pathologic diagnosis is essential in order to avoid erroneously upstaging the original primary, potentially leading to non-curative intent treatment. Hence, while a functional and anatomic study, such as the PET-CT, can initiate the speculation of a synchronous primary, further investigation must be sought especially in the absence of obvious widespread metastatic lesions.

## Conclusions

With patients living longer and widespread use of imaging, the rate of detection of multiple primary cancers is higher than before. Pursuing a prompt definitive diagnosis in these patients may enable effective treatment strategies.
